# Human Inhalation Exposure to Aerosol and Health Effect: Aerosol Monitoring and Modelling Regional Deposited Doses

**DOI:** 10.3390/ijerph17061923

**Published:** 2020-03-16

**Authors:** Hyeon-Ju Oh, Yoohan Ma, Jongbok Kim

**Affiliations:** 1 Department of Health & Environmental Science, Korea University, Seoul 02841, Korea; 2 Department of Materials Science and Engineering, Kumoh National Institute of Technology, 61 Daehak-ro (yangho-dong), Gumi, Gyeongbuk 39177, Korea; yoohanm@princeton.edu; 3 Department of Environmental Sciences, Rutgers, The State University of New Jersey, 14 College Farm Road, New Brunswick, NJ 08901, USA; 4 Andlinger Center for Energy and Environment, Princeton University, Princeton, NJ 08544, USA

**Keywords:** aerosol monitoring, modeling, inhalation exposure, bioaerosol, fractions of particulate matter

## Abstract

Since poor air quality affects human health in the short and long term, much research has been performed on indoor and outdoor aerosol exposure; however, there is a lack of specific data on the exposure and health risks of inhalable aerosols that contain bioaerosol in different environments of human life. To investigate the potential exposure to inhalable aerosols (in the monitoring of particulate matter (PM) based on R modeling, variations of PM depend on the ventilation system and bioaerosols based on size distribution) in various environments, the special viability and culturability of bioaerosols and their deposition doses in the respiratory system were evaluated. We conducted exposure assessments on inhalable aerosols in various indoor environments (childcare facilities, schools, commercial buildings, elderly and homes). The fractions of PM (PM_10_, PM_4_ and PM_2.5_) were investigated and, for the bioaerosol, the viability, culturability, inhalation daily dose and the deposited dose of the aerosol in the respiratory system were calculated to evaluate the human health effects. For two years, the distribution of the indoor PM concentration was high in all PM fractions in schools and commercial buildings, and low in the elderly and at homes. For airborne bacteria, the highest concentrations were shown in the childcare facility during the four seasons, while airborne fungi showed high concentrations in the buildings during the spring and summer, which showed significant differences from other investigated environments (between the buildings and elderly and homes: *p* < 0.05). The viability and culturability for the bioaerosol showed no significant difference in all environments, and the correlation between inhalable PM and bioaerosol obtained from the six-stage impactor showed that the coefficient of determination (R^2^) between coarse particles (PM_10–2.5_, the size of stage 2–3) and cultivable airborne bacteria ranged from 0.70 (elderly and homes) to 0.84 (school) during the summer season.

## 1. Introduction

The World Health Organization (WHO) announced that poor air quality can cause asthma, stroke, heart disease, and pregnancy complications [1,2,3]. Furthermore, there is evidence that short-term exposure to air pollution contributes to worsening mental health in children [4]. In other words, 1–2 days after a certain level of air pollution, children were more likely to go to the emergency room for mentally-related reasons [5]. In addition, it has already been confirmed that particles that have penetrated into the respiratory tract [6] interact with each other to affect blood cells as well as brain cells [5,7].

Today, outdoor pollutants, including carcinogens [3], have infiltrated into the living environment of elderly human beings, and their influence on regional air pollution is different according to the difference of living standards [1,8]. Outdoor air pollution is classified as a carcinogen or a cancer-causing substance [9]. Studies on exposure to particulate matter (PM) and health risks have been demonstrated through in vitro and in vivo studies [7,10,11], including epidemiological studies, and show that the hazards can cause vascular and brain diseases as well as respiratory diseases [12].

On the other hand, the biological fraction (bioaerosol) among the various components of PM [13] can have a significant impact on human health [6,13,14,15,16], including infectious diseases, acute toxic effects, allergies and cancer [14,17,18]. Many bacterial infectious diseases such as Legionellosis, Tuberculosis, Anthrax, etc., are due to chronic and even temporary exposure to bioaerosols [19]. This is because some aerosols, including bacteria and fungi, pollen, and viruses, remain in the atmosphere [13,15,16] and have a long atmospheric residence time, especially bacteria [15], including surviving and non-surviving bacteria [14,19], mainly due to the coarse particles (PM_10–2.5_) in the atmosphere.

In various environments (hospitals, animal warehouses, clean rooms, pharmaceutical facilities, etc.) [6,17], from birth to death, the presence of bioaerosols has been shown to impact human health, especially including multiple determinants (cooking, varied types of kitchen set) [6,15,20]. Exposure levels vary depending on the structural characteristics of houses, ventilation, location of the house [15,21,22], geographical conditions and exposure time [18]. During indoor and outdoor air quality monitoring, it was confirmed that outdoor harmful pollutants were penetrating indoors [21]. Inhalable aerosols that have penetrated the respiratory tract have different deposition rates [6,16] depending on the location of the respiratory system, and deposition levels and transport patterns provide important clues for assessing the potential health effects of aerosols [15,18].

Therefore, our study aims to (1) investigate exposure to indoor aerosols (fractions of PM and bioaerosols) in various environments for two years, (2) evaluate the viability and culturability for bioaerosols and the associated particulate matter, and (3) assess the inhalation risk associated with aerosols using the inhalation and deposition doses in the respiratory system.

## 2. Materials and Methods

### 2.1. Monitoring Campaign

The characteristics of the childcare facilities, schools, commercial buildings, elderly and homes are shown in Table 1. Sampling was carried out in three places—locations A, B and C—for each investigated facility, in a total of 15 places (in childcare facilities, schools, commercial buildings, elderly and homes) in Korea, and a total of 45 sampling points were determined by setting three sampling points for each site. The locations selected for the monitoring campaign were based on indoor air quality impact factors, such as the indoor air and outdoor pollutants mentioned in Table 1. The study was conducted in childcare facilities, schools, commercial buildings, elderly and homes, including several commercial shopping centers and general offices. The sampling locations were approximately 8–21 years old (Table 1) and located in a commercial area close to heavy traffic roads: 5000–9000 cars/day for A; 4000–7000 cars/day for B; and 3000–6000 cars/day for C.

The monitoring campaign was carried out in 45 sampling locations over four seasons (3/10/2015–12/19/2015 and 2/11/2016–12/19/2016) with continuous sampling in batches of 2–3 days followed by 1–2 days break for the maintenance of equipment.

**Figure 1:**
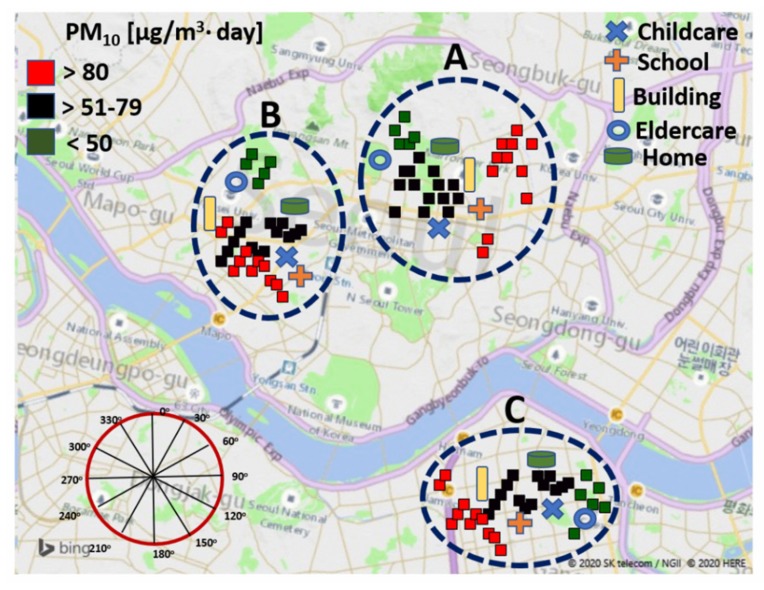
Sampling locations (A, B and C group for indoors of childcare, school, commercial building, elderly and homes for indoors and O for outdoor) with the plots of outdoor PM_10_ using R statistical software and Microsoft 3D maps.

The monitoring locations and their background PM_10_ concentrations are shown in Figure 1, and their plots were created using the R statistical software (Free Software Foundation, Inc., Boston, MA, USA) and Microsoft Bing maps in 3D format for Excel based on a three-dimensional (3D) data visualization tool.

### 2.2. PM Sampling

The fraction of indoor PM (PM_10_, PM_4_, PM_2.5_) was determined by Grimm 1.109 OPC (Grimm Technologies Inc., Douglasville, GA, USA) and, the outdoor particulate matters were measured using Grimm 1.109 OPC, which was installed at a distance of 100 m from each sampling location. In addition, temperature, relative humidity and wind speed were investigated every 4 h; temperature and relative humidity were measured for both indoor and outdoor locations, and wind speed was measured for outdoor locations. During real-time monitoring, the inlet of the sampler was placed about 1.2–1.5 m above ground at indoor and outdoor sampling points.

### 2.3. Bioaerosol Characteristics Associated with PM

For the culture-based bioaerosol analysis, the sampling of bioaerosols was performed using an Anderson sampler with a 400 (N) −0.25 mm holes (MAS-100 Eco, MERCK, Kenilworth, NJ, USA) operating pump (Buck, AP Buck, Inc., USA, flow rate: 100 L/min) to measure airborne bacteria and airborne fungi concentrations. The medium used was Tryptic Soy Agar (Hanil Komed CO., LTD., Seongnam, Korea) for airborne bacteria, and for the airborne fungi, SDAC (Sabouraud Dextrose Agar + Chloram, DIFCO., Swedesboro, NJ, USA) was used. Sampling was carried out three times at 15 min intervals in the morning and afternoon at all sampling locations. To prevent contamination between samples, sampler holes were cleaned with 70% alcohol before and after sampling. All samples taken at each sampling point were sealed with parafilm, placed in an ice-box and transported to the laboratory.

For the counting of the total bioaerosol, the bioaerosol field samples with BioSampler (SKC Inc., Eighty Four, PA, USA) were collected, and the airborne bacterial cells were determined by acridine orange epifluorescence microscopy using the Axioskop 20 (Carl Zeiss MicroImaging Inc., Thornwood, NY, USA) [1]; the airborne fungi were counted using direct light microscopy (Axioskop 20, Carl Zeiss MicroImaging Inc., Thornwood, NY, USA).

The cell viability was determined as a total cell population (living and dead cells). The viability allows us to assess healthy cells in field samples, and an increase in the viability indicates cell growth. The concentration of total bacterial and fungal cells was measured using a cell viability assay kit (Fluorometric-Green, Abcam plc, Cambridge, MA, USA) by flow cytometry (ThermoFisher Scientific, Waltham, MA, USA). We determined the cell viability as defined below:

(1) $$\mathrm{Viability}\;\mathrm{of}\;\mathrm{bioaerosol}\;\left(\%\right)=\frac{\mathrm{Concentration}\;\mathrm{of}\;\mathrm{living}\;\mathrm{cells}\left(\frac{\mathrm{cell}}{m^{3}}\right)}{\mathrm{Concentration}\;\mathrm{of}\;\mathrm{total}\;\mathrm{cells}\;\left(\frac{\mathrm{Cell}}{m^{3}}\right)}\times 100.$$

To count viable bioaerosols, culture-based analysis was performed; in particular, viable fungi can induce infectious disease in immunocompromised individuals. The culturability of bioaerosol was defined as the ratio of culturable bioaerosol concentration to total bioaerosol concentration, according to a previously published method [2].

(2) $$\mathrm{Culturability}\;\mathrm{of}\;\mathrm{bioaerosol}\;\left(\%\right)=\frac{\mathrm{Concentration}\;\mathrm{of}\;\mathrm{culturable}\;\mathrm{bioaerosol}\left(\frac{\mathrm{CFU}}{m^{3}}\right)}{\mathrm{Concentration}\;\mathrm{of}\;\mathrm{total}\;\mathrm{cells}\;\left(\frac{\mathrm{Cell}}{m^{3}}\right)}\times 100.$$

To assess the inhalation health effect associated with a bioaerosol, the viable index was calculated. A microbiologically viable index is used to indicate the number of total viable airborne bacteria and airborne fungi in the aerosol samples collected [3].

(3) Fviable=CCFUNparticles.

where C_CFU_ is the total culturable bioaerosol concentration (CFU/m^3^) and N_particles_ is the total amount of concentration (#/m^3^) measured using Grimm 1.109 OPC (Optical Particle Counter, Grimm Technologies Inc., Douglasville, GA, USA).

To evaluate the health effects for inhaled bioaerosols, the microbiological viable index was calculated using Equation (1) [14] and the concentration based on the size distribution was measured using a six-stage process (>7.0 µm, 4.7–7.0 µm, 3.3–4.7 µm, and 2.1–3.3 µm, 1.1–2.1 µm and 0.65–1.1 µm). After incubation for 48 h (for airborne bacteria) and 5 days (for airborne fungi), colonies were counted in TSA and MEA medium, respectively. In addition, in order to examine the correlation between particle matter concentration and bioaerosol concentration, we analyzed the correlation between coarse particles (PM_10–2.5_) and PM_2.5_ concentration with culture-based bioaerosol concentration using a six-stage impactor.

### 2.4. Inhalable Aerosol Health Effects

To assess the health effects associated with inhalable bioaerosols, a model [23,24,25] recommended by the US EPA [26] was applied, which estimates the average daily inhalable dose (ADD). The ADD for inhalable bioaerosols can be calculated by the following Equation (4).

(4) $$\mathrm{ADD}=\frac{C\times \mathrm{ET}\times \mathrm{EF}\times \mathrm{ED}}{\mathrm{AT}}$$

where ADD is the exposure concentration (cells/m^3^). C is the total bioaerosol concentration (Cells/m^3^), ET is the exposure time (8 h/day), EF is the exposure frequency (d/yr), ED is the exposure duration (yr) and AT is the average lifetime (h). However, for the parameter for ED, we considered the period according to the age-based activity period. In other words, for the childcare facility, the result of the survey (given in the Supplementary Materials) showed that children have an average age of 3 years for the average childcare facilities, 12 years is the average period of time for students to go to school, 30 years is the average of adult men and women living in the commercial building. When we consider the average life expectancy of 80 years, the period after retirement was applied as 35 years.

Human exposure modeling relates pollutant concentrations in the larger environmental media to pollutant concentrations in the immediate exposure media with which a human population has direct contact. Exposure may be quantified as the amount of the pollutant available at the boundary of the receptor organism per specified time period. From an exposure modeling standpoint, the principal goal is to estimate exposure as a function of both the relevant human factors and the measured or estimated pollutant concentrations in the contact or exposure media.

In this study, human exposure to air pollutants could include human factors such as all behavioral, sociological, and physiological characteristics of an individual. However, we have applied different indoor environments experienced in human life, and their exposure time was determined as 8 h (9 a.m.–5 p.m.) by the life patterns based on the survey (the survey results are not shown here).

Meanwhile, the evaluations of health effects based on PM were evaluated as the quantitative exposure assessment, which means the inhalable dose and deposited dose in the respiratory system. The “inhalable dose” means the dose that enters the human respiratory system and the “deposited dose” is deposited in a specific region of the respiratory system: the head airways (HA), the tracheobronchial region (TB), and alveolar region (AL). The inhalable and deposited dose was calculated by the equations of Nazarenko et al. [27].

### 2.5. Statistical Analysis

Since we have a large amount of data in different sampling locations, we used a one-way ANOVA. The mass concentrations were analyzed as a function of the investigated locations (i.e., childcare, schools, commercial building and elderly and homes) and seasons. For significant effects, all multiple pairwise comparisons were performed using the Holm–Sidak method with Sigmaplot 2011 (version 12.3, Systat Software Inc., San Jose, CA, USA). Data normality was assessed across all replicates using the Shapiro–Wilk test, and, if needed, a nonparametric Mann–Whitney rank-sum test was used to determine significantly different pairs. The latter comparison was performed for all location pairs measured with the same method.

## 3. Results

### 3.1. Outdoor and Indoor Aerosol Characterization

**Figure 2:**
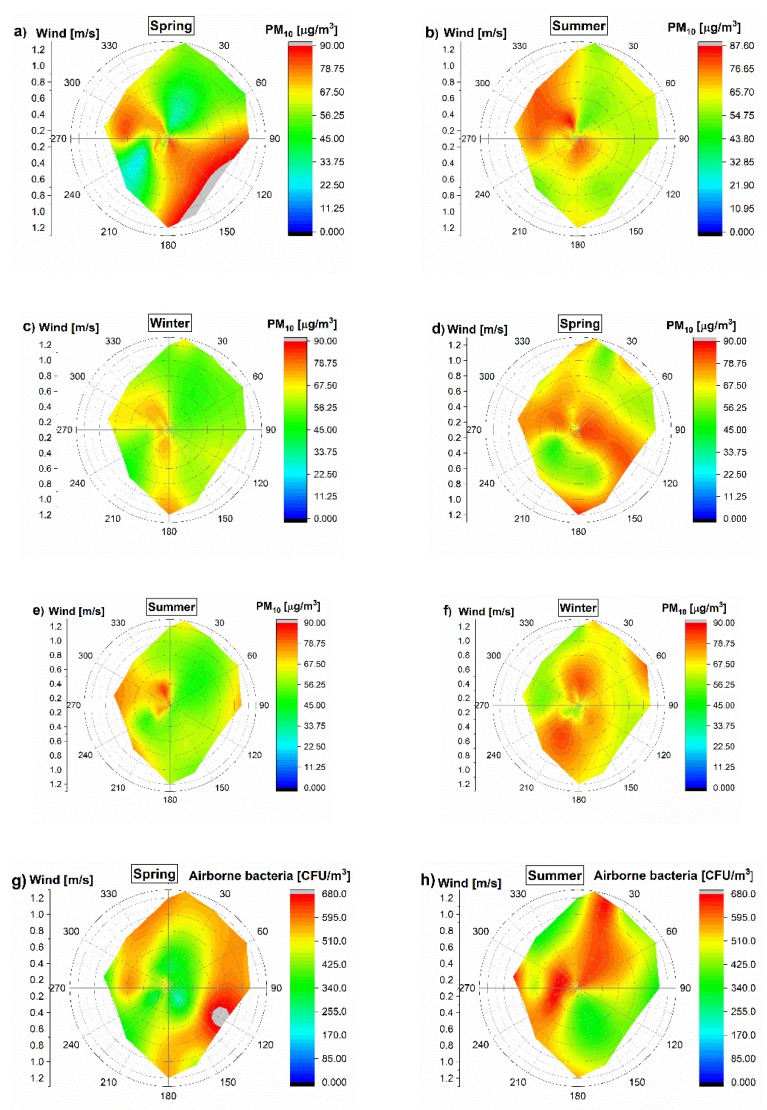

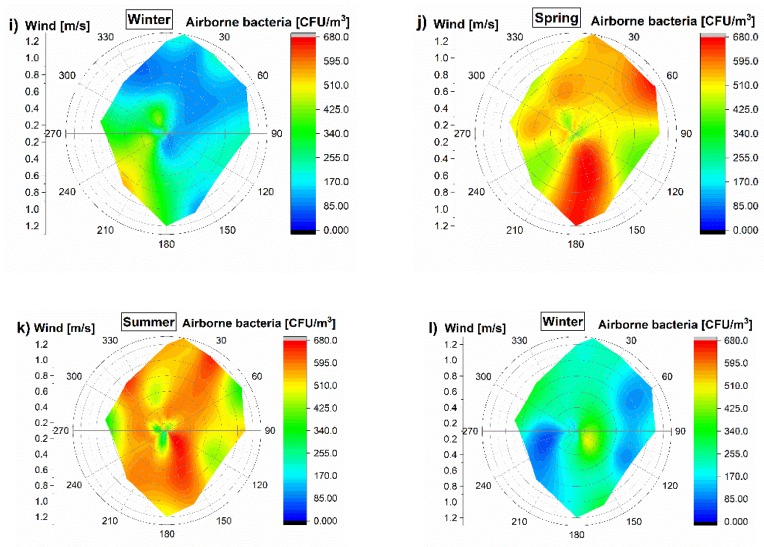
Polar contour maps for the concentrations of outdoor aerosol (PM_10_ and airborne bacteria) as a function of wind speed and direction. (**a**–**c**), (**g**–**i**) are investigated for 20 days in 2015; (**d**–**f**), (**j**–**l**) are investigated for 27 days in 2016. These data were obtained during the normal sunny days in each season.

Figure 1 presents the plots of PM_10_ concentration determined using the R statistical software. Outdoor PM_10_ concentrations are presented as three categories based on the concentration values. As shown in Figure 2, the average PM_10_ concentrations (24 h) with wind speed and direction were presented as well. The average PM_10_ concentrations ranged from 42.76 to 82.1 μg/m^3^ in all outdoor environments of the childcare, school, commercial building, elderly and homes.

We measured different inhaled particle fractions, including PM_10_, PM_4_ and PM_2.5_. The particle size fraction determines where the inhaled particles will deposit in the respiratory system, and the deposition site is a major factor determining the health effects, if any, of the deposited particles [25,26]. The majority of the PM_10_ mass will be deposited in the upper respiratory tract [27,28], while the majority of the PM_4_ particle mass will be deposited in the alveolar region [28].

**Figure 3:**
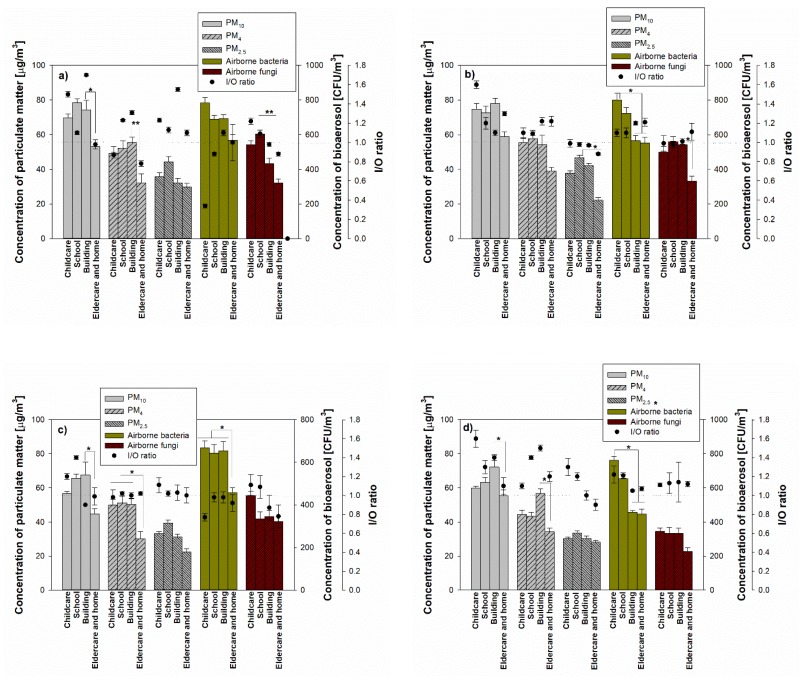
Concentrations of indoor aerosols (particulate matter (PM)_10_, PM_4_, PM_2.5_, airborne bacteria and airborne fungi) under different indoor environments during four seasons (**a**: spring, **b**: summer, **c**: autumn and **d**: winter).

In terms of the indoor PM concentration, the highest PM_10_ during the spring season was 78.5 ± 2.10 µg/m^3^ at school and the lowest was 53.2 ± 1.2 µg/m^3^ in the elderly and homes (Figure 3). For PM_4_ and PM_2.5_, the highest concentrations in commercial buildings and schools were 55.4 ± 3.2 µg/m^3^ and 44.3 ± 3.2 µg/m^3^, respectively, and the lowest concentrations were 32.2 ± 5.2 µg/m^3^ in the commercial building and 29.8 ± 2.1 µg/m^3^ for the elderly and homes. During summer, autumn and winter seasons, the highest PM concentrations are in schools, and PM concentrations observed from the elderly and homes showed the lowest values: 44.0–77.9 µg/m^3^ for average PM_10_, 30.2–57.8 µg/m^3^ for average PM_4_ and 22.2–29.8 µg/m^3^ for PM_2.5_.

For bioaerosol concentrations, airborne bacteria showed the highest concentrations in childcare facilities (799.4 ± 39.9 CFU/m^3^ in summer) in all seasons, while airborne fungi showed high concentrations (602.3 ± 24.5 CFU/m^3^) in school during summer. In addition, PM_4_ showed significant differences in concentrations in commercial buildings, elderly and homes in all seasons, and airborne bacteria and fungi also showed significant concentration differences in sampling locations (*p* < 0.05).

On the other hand, indoor air quality and occupant information relating to the investigated location were collected using supplemental material in the spring of 2016. The questionnaire was sent to the occupants (*n* = 342) in different locations (childcare facilities, schools, commercial buildings, elderly and homes). On average, two-thirds of the total personnel received the questionnaire. The difference between the indoor air quality with good and poor ventilation was very significant regarding the particulate matter and the smell of mold (*p* < 0.05) (not shown in this study). Interestingly, the higher-educated people occupying the commercial buildings and homes with good ventilation (124 respondents for commercial building and 46 respondents for homes) reported the least frequent environmental complaints. In addition, in indoor areas with poor ventilation (113 respondents), the occupants—regardless of working type and education—reported the most frequent environmental complaints and poor outdoor air quality.

### 3.2. Particle Size Distribution

**Figure 4:**
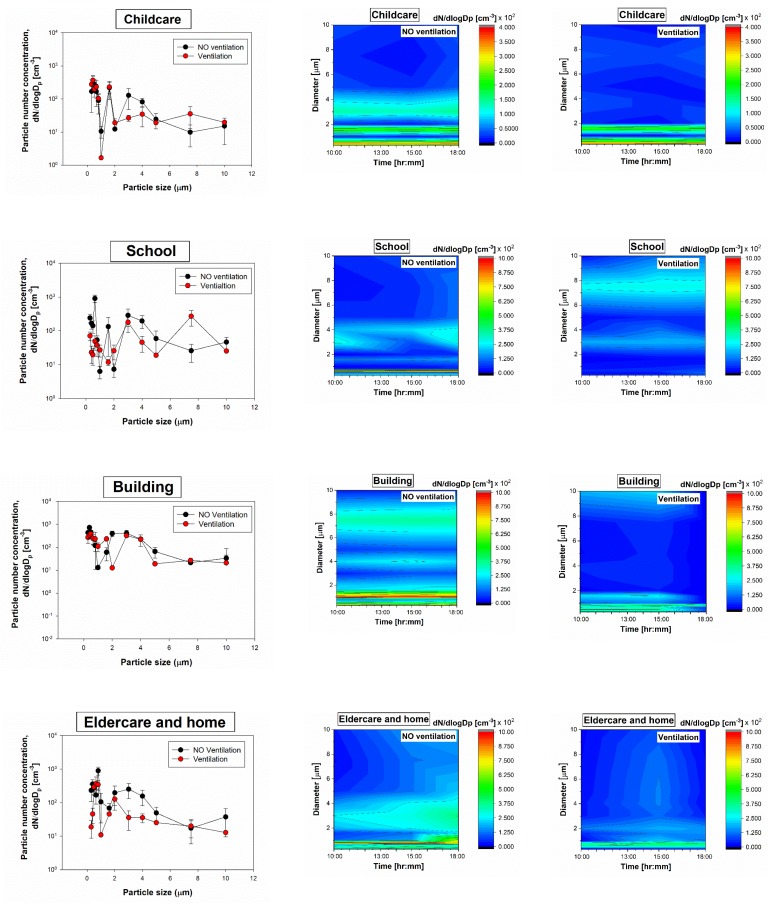
Size distributions of indoor particles in different places (childcare, school, commercial building, elderly facility and homes) and their variation with time as a function of ventilation. The data present averages of three repeats.

The normalized particle count distributions for all indoor environments were measured with a Grimm 1.109 OPC. The highest particle count concentrations were observed for the small size fractions and respirable fractions (<4 µm), and the lowest particle count concentrations were observed for the largest particles (Figure 4). The color contour maps show the significant differences for respirable-size particle concentrations (especially <2 µm) between particle sizes and between investigated locations.

When the ventilation systems were not operated, the particle count concentrations ranged from 9.9 particles/cm^3^ (childcare facility) to 9.1 × 10^2^ particles/cm^3^ (school), and the concentrations decreased with increasing particle size, while during the ventilation system operation, the particle count concentrations ranged from 1.6 particles/cm^3^ (childcare facility) to 3.8 × 10^2^ particles/cm^3^ (commercial building).

Overall, the concentration profiles for all indoor environments were different. As might be expected, the indoor particle concentrations when only ventilation was not operated were lower by 1–3 orders of magnitude than when the ventilations were operated.

The proper design, operation and maintenance of the ventilation system is essential in providing indoor air that is free of harmful concentrations of pollutants. Sources of indoor air pollution are caused by an accumulation of contaminants that come primarily from inside the commercial building, although some originate outdoors. Based on the health effect associated with the ventilation system, the harmful pollutants from a variety of sources can contribute to commercial building-related illnesses, which have clearly identifiable causes. The Heating, ventilation, and air conditioning (HVAC) system, when improperly operated or maintained, can contribute to sick building syndrome (SBS).

Even though there are various pollutant sources in different indoor environments applied in this study, we have focused on the HVAC systems only.

### 3.3. Bioaerosol Viability, Culturability and Relationship between PM and Bioaerosol

**Figure 5:**
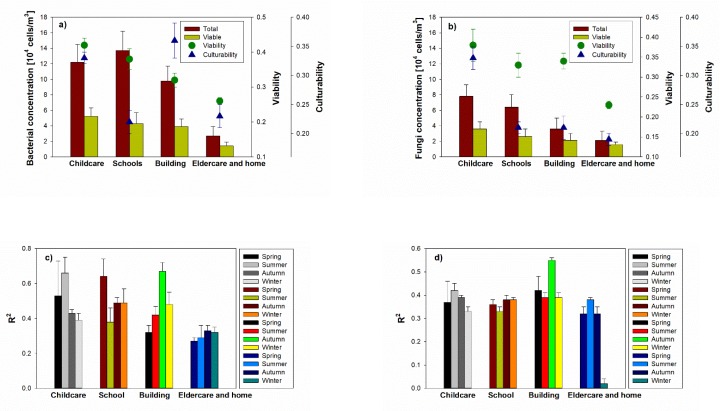
Concentrations of total bioaerosols, viability, culturability and the relationship between PM and viable bioaerosol observed in different indoor environments (childcare facility, school, commercial building and elderly and homes). (**a**,**b**) calculated viability and culturability for bacteria cells and fungal cells, respectively and (**c**,**d**) a linear relationship with coefficient of determination (R^2^) between viability and culturability for bacterial cells and fungal cells, respectively.

Their viabilities were 0.26% (in elderly and homes) to 0.42% (in childcare facilities) for airborne bacteria, and 0.23% (in elderly and homes) to 0.38% (in childcare facilities) for airborne fungi. As expected, the viability shows high values in the childcare facility with high concentrations of bioaerosol (Figure 5), but there was no significant difference in the viability of both airborne bacteria and fungi between indoor environments. In terms of the culturability, the airborne bacteria and fungi reached 0.22% (in the schools) to 0.36% (in the commercial building) and 0.19% (in the elderly and homes) to 0.33% (in the childcare facilities), respectively. There were no significant differences in culturability between indoor environments for either fungi or bacteria.

Meanwhile, there are differences both for the schools and commercial buildings between viability and culturability (*p* < 0.001). Many indoor bioaerosols originate both indoors and outdoors. However, the operation of the ventilation system can affect the bioaerosol concentration, with the variation being due to the penetration of outdoor and indoor source such as human activities (i.e., coughing, human skin, and sneezing), resulting in the differences between the viability and culturability of both schools and commercial buildings.

Relatively strong correlations (max: 0.64, 0.66 and 0.67) were found in all investigated indoor environments (except elderly and homes) depending on the season. The relative higher fidelity of correlation to PM over the four seasons may be due to the ventilation operation, due to indoor and other outdoor pollution sources. The enrichment of organic particles of PM_10_ in the indoor air may be explained by the presence of human skin particles, as they showed the same morphology as skin scrapings collected directly from healthy individuals. Since organic carbon is the major source of indoor PM_10_, human keratin from the skin was reported to contribute dominantly to indoor PM_10_ in schools, originating from the human personal cloud.

### 3.4. Concentrations of Bioaerosols Using Six-Stage Impactor and Relationship between PM and Culturable Aerosol

**Figure 6:**
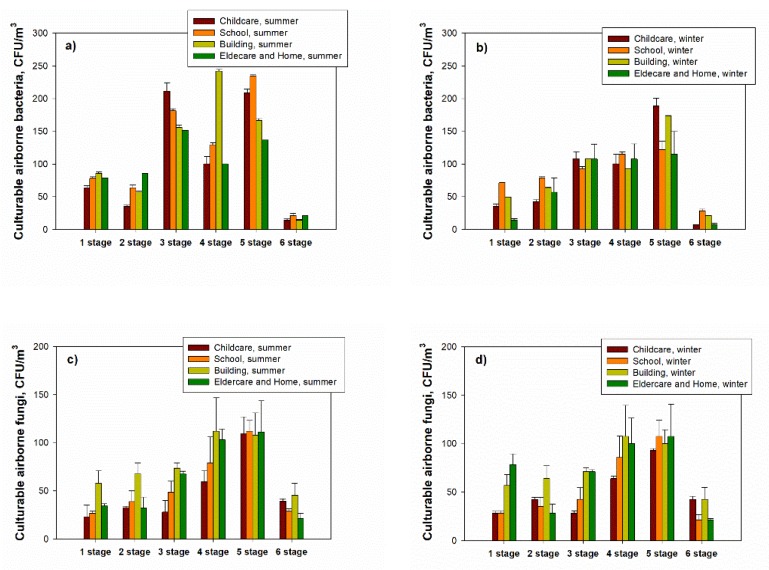
Culturable airborne bacteria (**a**,**b**) and fungi (**c**,**d**) during summer and winter seasons in different environments (childcare facilities, schools, commercial buildings and elderly and homes) using the six-stage Anderson impactor.

The bacterial concentrations during the summer season were highest at stage 3 (3.3–4.9 μm) at all measurement sites (except for the commercial building) and minimum at stage 6 (0.65–1.1 μm); for the airborne fungi, the highest concentration was reached at stage 5 (1.1–2.1 μm) at all indoor investigated locations (except for commercial buildings), and the lowest concentration was shown at stage 6 (Figure 6).

**Figure 7:**
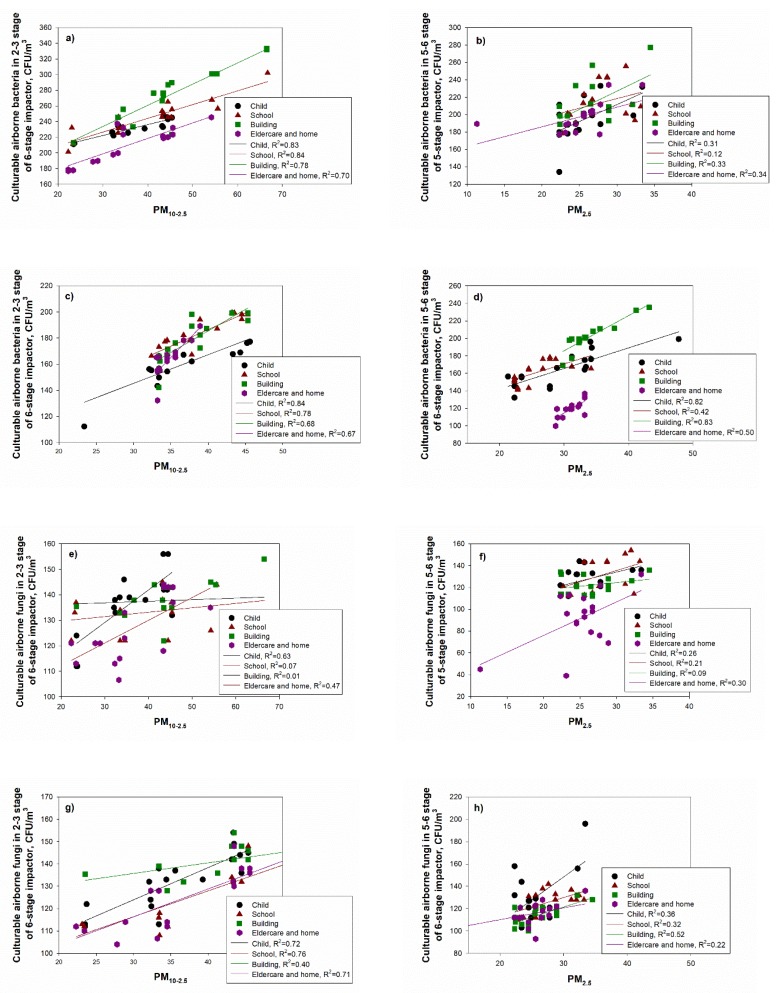
Correlation between PM concentrations and culturable bioaerosol. (**a**–**d**) airborne bacteria during spring, summer, autumn and winter, respectively. (**e**–**h**) airborne fungi during spring, summer, autumn and winter, respectively.

The relationship between PM concentrations and bioaerosols was presented as the coefficient of determination (R^2^) for PM_2.5_, PM_2.5–10_ and PM_10_ concentrations and airborne and fungi concentrations at each stage based on the six-stage impactor. During the summer season, the coefficient of determination (R^2^) between coarse particles (PM_10–2.5_) and culturable airborne bacteria at stages 2–3 (3.3–7.0 µm) ranged from 0.70 (for the elderly and homes) to 0.84 (for schools) (Figure 7). For PM_2.5_ and culturable airborne bacteria at stages 5–6 (0.65–2.1 µm), values ranged from 0.12 (for school) to 0.34 (for the elderly and homes). During the winter, R^2^ values for coarse particles (PM_10–2.5_) and culturable airborne bacteria in stages 2–3 ranged from 0.67 (for the elderly and homes) to 0.84 (for the childcare facility), while values for PM_2.5_ and culturable airborne bacteria in stages 5–6 ranged from 0.42 (for schools) to 0.82 (Figure 7).

### 3.5. Assessment of Health Effects for Inhalable Bioaerosol

**Figure 8:**
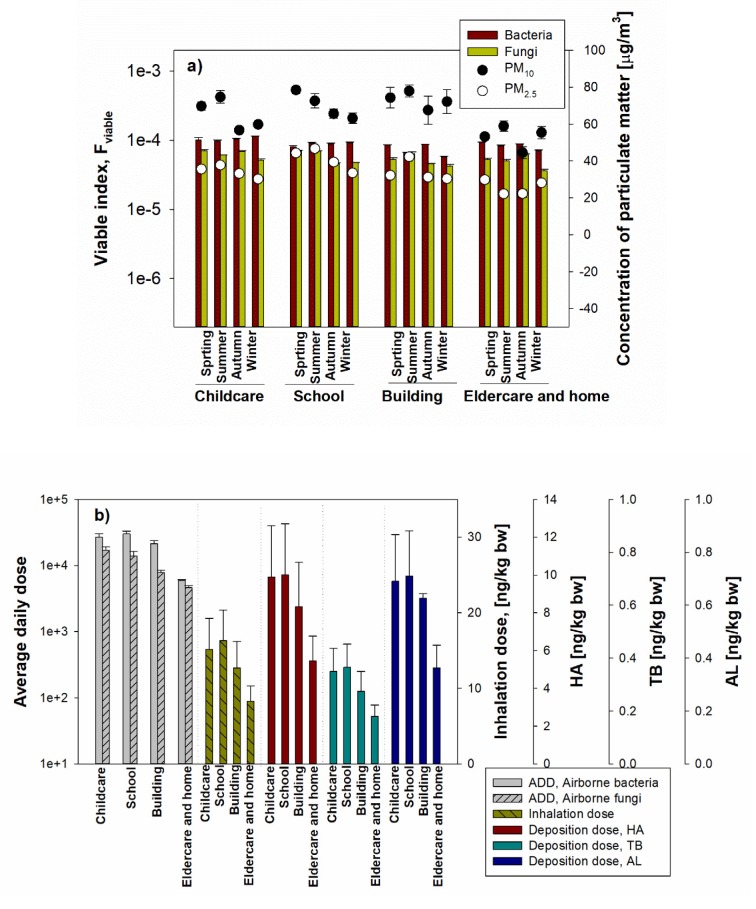
Microbiological viable index of total particles in different environments over four seasons (**a**), the average daily dose (ADD) (**b**) based on the total microbes (airborne bacteria and fungi) and inhalation/deposition dose based on the particle number concentrations. Deposited mass was calculated for the head airways (HA), the tracheobronchial (TB), the alveolar (AL) regions. The error bars represent one standard deviation.

Using the equation applied by Xu and Yao [14], the microbiological viable index for bioaerosol was calculated in the different environments. As shown in Figure 8a, in all measured facilities, the viable indexes of bacteria in all investigated locations were higher than those of airborne fungi, but there was no statistically significant difference. In addition, each viable index showed similar concentration levels (1 × 10^4^) in all environments. These values are higher than the calculated values (from 1 × 10^2^ to 1 × 10^4^) for the various environments (hotel rooms, student dormitories, laboratories, restaurants, outdoor environments) [14], which means that the viable microbial load was high in the measured environments. In addition, when comparing the viable index and PM (PM_10_, PM_2.5_) concentration, there was a significant difference in the PM concentration in the indoor environment of the target facility, but the viable index was not statistically significant. Compared to airborne bacteria, lower viable indexes have been observed for airborne fungi due to the lower cultivable fungal aerosol concentrations, but in general, the viable index of airborne bacteria and airborne fungi are similarly high, which means that an inhalation risk existed for all investigated environments.

To evaluate the health effects associated with bioaerosols, we have applied a model recommended by the US EPA, which uses the average daily dose (ADD) rates of inhalation by considering the complexity of bioaerosols. Both for bacteria and fungi, the calculated ADD ranged from 1 × 10^3^ cells/m^3^ to 1 × 10 × 10^4^ cells/m^3^ (Figure 8b).

Inhalable aerosols in the various environments were calculated as the mass dose of inhaled aerosol and also as the mass dose of aerosol deposited in the three regions of the human respiratory system: the head airways, the tracheobronchial and the alveolar regions. Furthermore, we compared the deposition in different regions of the respiratory system in the different exposed environments during the entire human lifetime.

In this study, the inhalation dose and deposition dose were calculated using the number of concentrations obtained by Grimm 1.109 OPC based on the International Commission on Radiological Protection (ICRP) model [27,28]. The ICRP model can be applied to aerosol particles and vapors from an atomic size up to very coarse aerosols (with an activity median aerodynamic diameter of 100 µm) in all practical conditions [6]. This model is designed to predict regional deposition based on the respiratory system in different subjects, including age and sex.

For all investigated locations during the human lifetime, the inhalation dose calculated as the mass of inhaled PM per kilogram of body weight is shown in Figure 8b. The inhalation dose was converted to mass concentration based on the number concentration of the aerosol size fractions up to PM_0.3_–PM_20_, applying the selected aerosol fraction in Grimm 1.109 OPC and applied to the ICRP model equation. Inhalation doses for the measured childcare facility, school, commercial building, elderly and homes were 15.2 ± 4.12 ng/kg·bw, 16.3 ± 4.08 ng/kg·bw, 12.69 ± 3.53 ng/kg·bw and 8.32 ± 1.98 ng/kg·bw, respectively.

On the other hand, based on the fine dust inhalation, the deposition doses in the respiratory tract areas HA, TB, and AL were calculated (Figure 8b): 5.46–10.01 ng/kg·bw (90.2–91.0%) for HA, 0.18–0.37 ng/kg·bw (2.9–3.3%) for TB and 0.36–0.71 (6.0–6.8%) in AL. The deposited dose in HA showed the highest deposition rate of 91% in the elderly facility and homes, and less than 10% in TB and AL; the highest value (3.3%) was present in schools for TB and (6.8%) in commercial buildings for AL.

## 4. Discussion

This study shows that the levels of exposure to aerosols (based on size-selection) are associated with health effects determined by the deposited dose and average daily dose in the respiratory system. We investigated the fraction of PM and bioaerosols in the different indoors; the evaluation of bioaerosol characteristics and the relationship between PM and bioaerosols was based on the size-selective concentration. Furthermore, the viability and culturability for bioaerosol were also considered as their aged-based aerosol deposition in the respiratory system along with the bioaerosol viable index contributing to health effects.

The present study gives the seasonal variations of PM_10_, PM_4_ and PM_2.5_ concentrations for varied indoor environments in urban areas; for PM and bioaerosols, concentrations considered wind speed along with an evaluation of the viability and culturability of airborne bacteria and fungi in different indoor environments.

Exposure to roadway emissions is an emerging area of research because of recent epidemiological studies reporting an association between living within a few hundred meters of high-traffic roadways and adverse health effects. The air quality impact of roadway emissions has been studied in a number of field experiments, most of which have not fully considered the impact of wind direction on near-road concentrations. This paper examines the role of wind direction by using a dispersion aerosol concentration to analyze data.

We found that wind direction is an important variable in characterizing exposure to roadway emissions. Under stable conditions, the near-surface concentrations at receptors up to 200 m from the road increased with wind angle before dropping off at angles close to parallel to the road. Only for pollutants with a short lifetime does the maximum concentration occur when the wind direction is normal to the road. We also show that current investigated aerosol concentrations (PM and bioaerosols) are reliable tools for interpreting observations and for evaluating predictive atmospheric models on all spatial and temporal scales for forecasting the nation′s air quality.

A past study showed that weather patterns, even the lowest wind speeds, are associated with PM_2.5_ concentration from October until April, and the measurement of wind speed and wind direction is important in air quality monitoring [11]. Since wind speed and direction can help identify the location of the source of the pollution and also provide a better overall picture of occurrences in the air, for aerosol monitoring, the application of wind speed could be helpful in developing emission control policies and regulations for air quality improvements [29].

While the total cells (living and dead cells) mainly move when attached to coarse particles (PM_2.5–10_), the viability decreased from their source during long-range transport. The viability and culturability determined in this study showed the highest values in the childcare facility; however, there were no significant differences between childcare facilities and schools/buildings, indicating the same pollutant sources and long-term accumulation without transport and/or movements of bioaerosol. The results show that children’s potential health risks to bioaerosols may be present over time in children’s homes and schools; particularly, high levels of viability indicate health-related exposure to bioaerosols.

The childcare facilities have led the way in demonstrating many of the links between environmental exposures and health outcomes. Some bioaerosols could also be adsorbed to the inhalable aerosols and transported in environments that have potential pollutant sources [16,30,31]. This study showed that PM concentrations, especially respirable-sized PM, are related to the culturable bioaerosol based on the size-selective concentration. In addition, the coefficient of determination between PM and bioaerosol (Figure 7) was over 0.7, and PM_2.5_ was even correlated with bioaerosols in some environments (childcare facilities and commercial buildings) (R^2^ = 0.8), indicating that a higher PM concentration could lead to the increase of bioaerosol concentration, causing respiratory diseases.

The viability associated with the concentrations of bioaerosols are due to indoor sources such as children’s activity, for which skin fragments [2,3], classroom cleaning, and cooking could be a major source of the bacteria at childcare facilities. This is also consistent with previous findings that correlate existing indoor air quality and viability [4].

Due to the size-related properties of aerosols (especially respirable-sized particles), the deposited respirable doses in the respirable system have shown potential problems, including invasion, retention and mobility in the human body. That is, in various parts of the respiratory system and the gastrointestinal tract [6,32], they can be transported to other organs of the rodent body through the bloodstream and the nervous system [7,33].

The rates of ADD inhalation were calculated according to the equation selected by Chen and Carter [23] and for the exposure duration (yr), life cycle activity/exposure was applied differently to the childcare facilities, schools, commercial buildings, elderly and homes. The health effects for the bioaerosol were evaluated by ADD values, while PM was evaluated as the inhalation dose and deposited dose.

It is often difficult to determine the extent to which the environment contributes to human health. In addition, the control of respirable-sized aerosols in PM and bioaerosols should be required considering the exposure environments, age, and a potential source of pollution [4,34,35]. Moreover, the viability could be an important tool in the evaluation of the health effect when indoor pollutant factors and/or sources were considered.

As shown in different indoor environments (childcare facilities, schools, commercial buildings, elderly and homes), even though we could not include all ages (newborns and pre-school individuals were not included), the health effects evaluated in this study could be a challenge for extensive investigation with the individual characteristics based on age and indoor and outdoor pollutant sources. In this study, we could not control the various indoor and outdoor factors regarding pollutant sources. However, we focused more on the fractions of culturable bioaerosol associated with respirable-size PM for a given size range in different indoor environments and the adverse health consequences of environmental exposures along with the deposited doses of aerosol.

Our results show that human inhalation exposure to aerosol and health effects were associated with exposure assessment considering the size-selective aerosol. This work fills the literature gaps regarding the multiple regression analysis results that varied with PM and bioaerosols based on the size-selective and viable/culturable bioaerosol investigated in different indoor environments.

## 5. Conclusions

Human exposure to aerosol and health effects based on the size-selective aerosol concentration were investigated. This study shows that size-selective aerosol concentrations can be used as a basic indicator of health risks and suggests a health-effect assessment based on aerosol characteristics (aerosol size and bioaerosol viability). The exposure to inhalable (bio) aerosols in various environments of human life varies widely, and thus the health risks can vary greatly. Therefore, exposure to inhalable aerosol will be required to obtain quantitative exposure data for a variety human environments and necessary for considering the age and potential source of pollution. Moreover, the viability could be an important tool in the evaluation of the health effect when indoor pollutant factors and/or sources were considered.

Ongoing aerosol exposure data and health impact assessments will be needed. In particular, continuous exposure studies of PM and bioaerosols are necessary for inhalable aerosol exposure based on size-selective aerosols, considering health effects and pollutant management in childcare facilities and schools. Our study suggests that exposure simulation and quantification of particle size, as well as mass-based and culture-based airborne particle fractions, could be part of an exposure toolbox for assessing human health.

## Figures and Tables

**Table 1:** Characteristics of sampling sites in all investigated environments.

Type	Sampling Site	Sampling Duration (Days)	Number of Samples	Area (m^2^), Mean (SD)	Potential Pollutant Source	Building Age	Number of People	
							Adult	Children
Childcare facility	CH-A (1, 2, 3)	148	231	56 (13)	Outdoor Cooking Cleaning	8	5	34
	CH-B (1, 2, 3)	150	336	59 (4)		11	4	36
	CH-C (1, 2, 3)	136	231	52 (9)		9	5	38
School	SC-A (1, 2, 3)	148	102	52 (6)	Outdoor Cooking Cleaning	11	6	78
	SC-B (1, 2, 3)	142	213	59 (12)		8	6	76
	SC-C (1, 2, 3)	148	219	54 (8)		12	6	78
Building	BU-A (1, 2, 3)	132	123	52 (6)	Outdoor Cleaning	21	54	-
	BU-B (1, 2, 3)	126	186	55 (3)		15	53	-
	BU-C (1, 2, 3)	148	273	62 (2)		14	48	-
Elderly	EL-A (1, 2, 3)	136	123	53 (4)	Outdoor Cooking Cleaning	8	32	-
	EL-B (1, 2, 3)	116	123	55 (6)		9	31	-
	EL-C (1, 2, 3)	126	102	54 (4)		9	29	-
Home	HO-A (1, 2, 3)	120	108	49 (3)	Outdoor Cooking Cleaning	12	8	-
	HO-B (1, 2, 3)	142	129	45 (3)		14	11	-
	HO-C (1, 2, 3)	136	165	48 (2)		7	16	-

CH: childcare facility, SC: school; BU: commercial building, EL: elderly, HO: homes. A, B and C: each investigated group.

## Supplementary Materials

The following are available online at https://www.mdpi.com/1660-4601/17/6/1923/s1, File S1: the result of the survey.
