# Supplementary material for: Human Inhalation Exposure to Aerosol and Health Effect: Aerosol Monitoring and Modelling Regional Deposited Doses
Source: Int J Environ Res Public Health. 2020 Mar 16;17(6):1923. doi: 10.3390/ijerph17061923 (PMC7142517; doi:10.3390/ijerph17061923)
Supplement: Supplementary file 1 [file ijerph-17-01923-s001.pdf]

Supplemental Material

File S1. the result of the survey.

SURVEY

Building	Type	
	Address	
	Age	
	Area	m^2
	Total floor	
	Windows	Number of windows we can open:<br>Area: m^2
	Number of people	Student:<br>Teacher:
People	Age	
	Sex	
	Education	
	Working type	
	Pattern of life	
	Exercise	
	Allergy	
	Opinion of indoor air quality	
	Others	

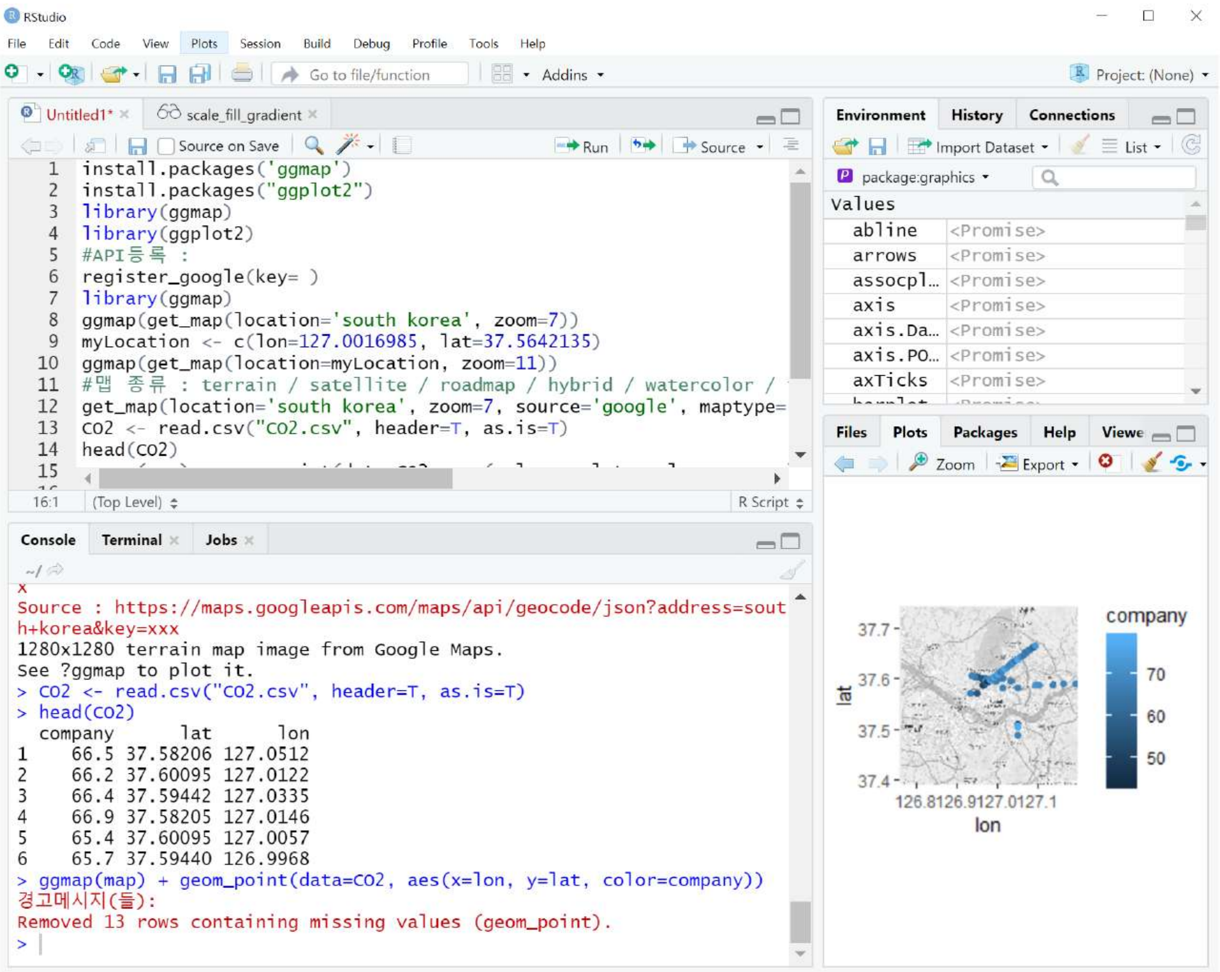

RStudio
File Edit Code View Plots Session Build Debug Profile Tools Help
Go to file/function
Addins
Project: (None)
Untitled1*
scale_fill_gradient
Source on Save
Run
Source
install.packages('ggmap')
install.packages("ggplot2")
library(ggmap)
library(ggplot2)
#API등록 :
register_google(key=)
library(ggmap)
ggmap(get_map(location='south korea', zoom=7))
myLocation <- c(lon=127.0016985, lat=37.5642135)
ggmap(get_map(location=myLocation, zoom=11))
#맵 종류 : terrain / satellite / roadmap / hybrid / watercolor /
get_map(location='south korea', zoom=7, source='google', maptype=
co2 <- read.csv("co2.csv", header=T, as.is=T)
head(co2)
16:1
(Top Level)
R Script
Console
Terminal
Jobs
~/
x
Source : https://maps.googleapis.com/maps/api/geocode/json?address=south+korea&key=xxx
1280x1280 terrain map image from Google Maps.
See ?ggmap to plot it.
> co2 <- read.csv("co2.csv", header=T, as.is=T)
> head(co2)
company lat lon
1 66.5 37.58206 127.0512
2 66.2 37.60095 127.0122
3 66.4 37.59442 127.0335
4 66.9 37.58205 127.0146
5 65.4 37.60095 127.0057
6 65.7 37.59440 126.9968
> ggmap(map) + geom_point(data=co2, aes(x=lon, y=lat, color=company))
경고메시지(들):
Removed 13 rows containing missing values (geom_point).
>
Environment
History
Connections
Import Dataset
List
package:graphics
Values
abline <Promise>
arrows <Promise>
assocpl... <Promise>
axis <Promise>
axis.Da... <Promise>
axis.PO... <Promise>
axTicks <Promise>
Files
Plots
Packages
Help
Viewer
Zoom
Export
company
70
60
50
37.7
37.6
37.5
37.4
lat
126.8126.9127.0127.1
lon

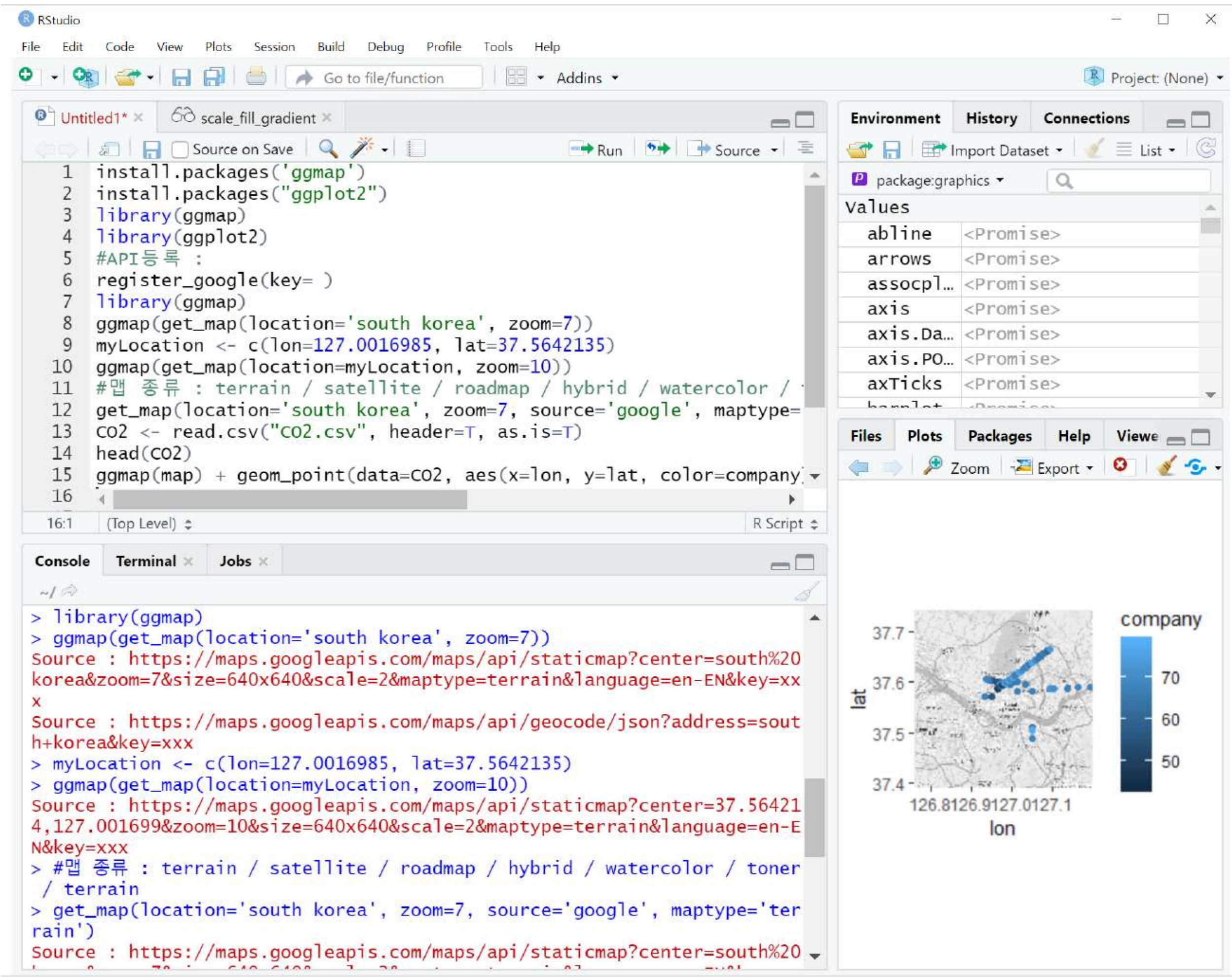
RStudio
File Edit Code View Plots Session Build Debug Profile Tools Help
Go to file/function
Addins
Project: (None)
Untitled1*
scale_fill_gradient
Source on Save
Run
Source
install.packages('ggmap')
install.packages("ggplot2")
library(ggmap)
library(ggplot2)
#API등록 :
register_google(key=)
library(ggmap)
ggmap(get_map(location='south korea', zoom=7))
myLocation <- c(lon=127.0016985, lat=37.5642135)
ggmap(get_map(location=myLocation, zoom=10))
#맵 종류 : terrain / satellite / roadmap / hybrid / watercolor /
get_map(location='south korea', zoom=7, source='google', maptype=
CO2 <- read.csv("CO2.csv", header=T, as.is=T)
head(CO2)
ggmap(map) + geom_point(data=CO2, aes(x=lon, y=lat, color=company)
16:1
(Top Level)
R Script
Console
Terminal
Jobs
~/
> library(ggmap)
> ggmap(get_map(location='south korea', zoom=7))
Source : https://maps.googleapis.com/maps/api/staticmap?center=south%20korea&zoom=7&size=640x640&scale=2&maptype=terrain&language=en-EN&key=xxx
Source : https://maps.googleapis.com/maps/api/geocode/json?address=south+korea&key=xxx
> myLocation <- c(lon=127.0016985, lat=37.5642135)
> ggmap(get_map(location=myLocation, zoom=10))
Source : https://maps.googleapis.com/maps/api/staticmap?center=37.564214,127.001699&zoom=10&size=640x640&scale=2&maptype=terrain&language=en-EN&key=xxx
> #맵 종류 : terrain / satellite / roadmap / hybrid / watercolor / toner / terrain
> get_map(location='south korea', zoom=7, source='google', maptype='terrain')
Source : https://maps.googleapis.com/maps/api/staticmap?center=south%20
Environment
History
Connections
Import Dataset
List
package:graphics
Values
abline <Promise>
arrows <Promise>
assocpl… <Promise>
axis <Promise>
axis.Da… <Promise>
axis.PO… <Promise>
axTicks <Promise>
Files
Plots
Packages
Help
Viewe
Zoom
Export
company
70
60
50
37.7
37.6
37.5
37.4
lat
126.8126.9127.0127.1
lon
